# C2 Pedicle Screw Placement: A Novel Teaching Aid

**DOI:** 10.7759/cureus.630

**Published:** 2016-06-04

**Authors:** Olaide Ajayi, Marc Moisi, Jens Chapman, Rod J Oskouian, R. Shane Tubbs

**Affiliations:** 1 Department of Neurosurgery, Loma Linda University Medical Center; 2 Seattle Science Foundation; 3 Neurological Surgery, Wayne State University; 4 Neurosurgery, Swedish Neuroscience Institute; 5 Neurosurgery, Complex Spine, Swedish Neuroscience Institute; 6 Neurosurgery, Seattle Science Foundation

**Keywords:** c2 pedicle screw, teaching aid, technique

## Abstract

The C2 pedicle screw is more biomechanically stable and provides patients with increased postoperative range of motion in comparison to other methods of C2 fixation. However, as a result of the proximity of the C2 pedicle to the transverse foramen, there is a considerable risk of intraoperative morbidity due to vertebral artery injury laterally or vertebral canal breach medially. Other than the use of cadavers for the demonstration and practice of C2 pedicle screw placement, there are currently few other readily available teaching aids for the training of residents and fellows. Herein, we describe a simple and cost effective modality for the demonstration, evaluation, and practice of C2 pedicle screw placement in a laboratory setting.

## Introduction

Atlantoaxial instability may occur as a result of trauma, tumors, congenital malformations, or inflammatory conditions of the upper cervical spine [1-2]. Fixation techniques for the treatment of atlantoaxial instability have evolved from spinous process wiring techniques first described by Mixter and Osgood in 1910 [3-4], to laminar wiring described by Gallie in 1939 [3, 5] and C1-2 laminar wiring described by Brooks and Jenkins in 1978. Dickman and Sonntag further modified the posterior wiring technique in 1991 [3, 6].

Posterior C1-C2 transarticular screws and C1-lateral mass screws with C2 pars screws are some of the more recently developed methods of C1-C2 fixation. The C2 pedicle screw was first described by Goel, et al. in the 1980s as part of a plate and screw construct that was utilized for posterior C1-2 fixation [3, 7-8]. This technique has been further modified, with a variety of poly-axial screws and top-loading rods now widely available for C2 pedicle fixation.

The choice of any of these methods of fixation over another are influenced by anatomic constraints or variations, surgeon’s preference and/or experience [1, 9] as well as comparative knowledge of the strengths and limitations of each construct.

### Background

The C2 pedicle screw technique has numerous advantages over other methods of C2 fixation such as the C1-2 transarticular screw technique, the C2 lateral mass screw as well as the posterior wiring techniques. These advantages include increased range of motion postoperatively as well as stronger biomechanical stability. Based on a biomechanical study by Lehman, et al., C2 pedicle screws generated a greater insertional torque and pull-out strength in comparison to lateral mass, pars, and laminar screws. [10]

C2 pedicle screw placement is technically demanding and the precise and exact three-dimensional understanding of the anatomy of the region and vertebral artery is mandatory [3]. Apart from utilizing cadavers to demonstrate the placement of C2 pedicle screws and the avoidance of complications (such as vertebral artery injury or spinal cord injury due to medial breach of the pedicle screw into the vertebral canal) during the procedure, there are currently few teaching aids for the training of residents and fellows.

Herein, we describe an easily applicable teaching aid that utilizes dried human C2 vertebrae to demonstrate the procedure while highlighting the avoidance of complications.

## Technical report

The C2 pedicle is the portion of the C2 vertebra anterior to the pars, connecting the dorsal elements with the vertebral body. The entry point for the C2 pedicle screw is usually 5 mm rostral and 1 mm medial to the inferomedial aspect of the inferior articulating surface of C2. The screw is placed with 15-25 degrees of medial angulation with the thick medial wall of the C2 pedicle helping to redirect the screw if necessary, to prevent medial wall breakout and entry into the spinal canal. [3]

## Discussion

### Methods

**Figure 1:**
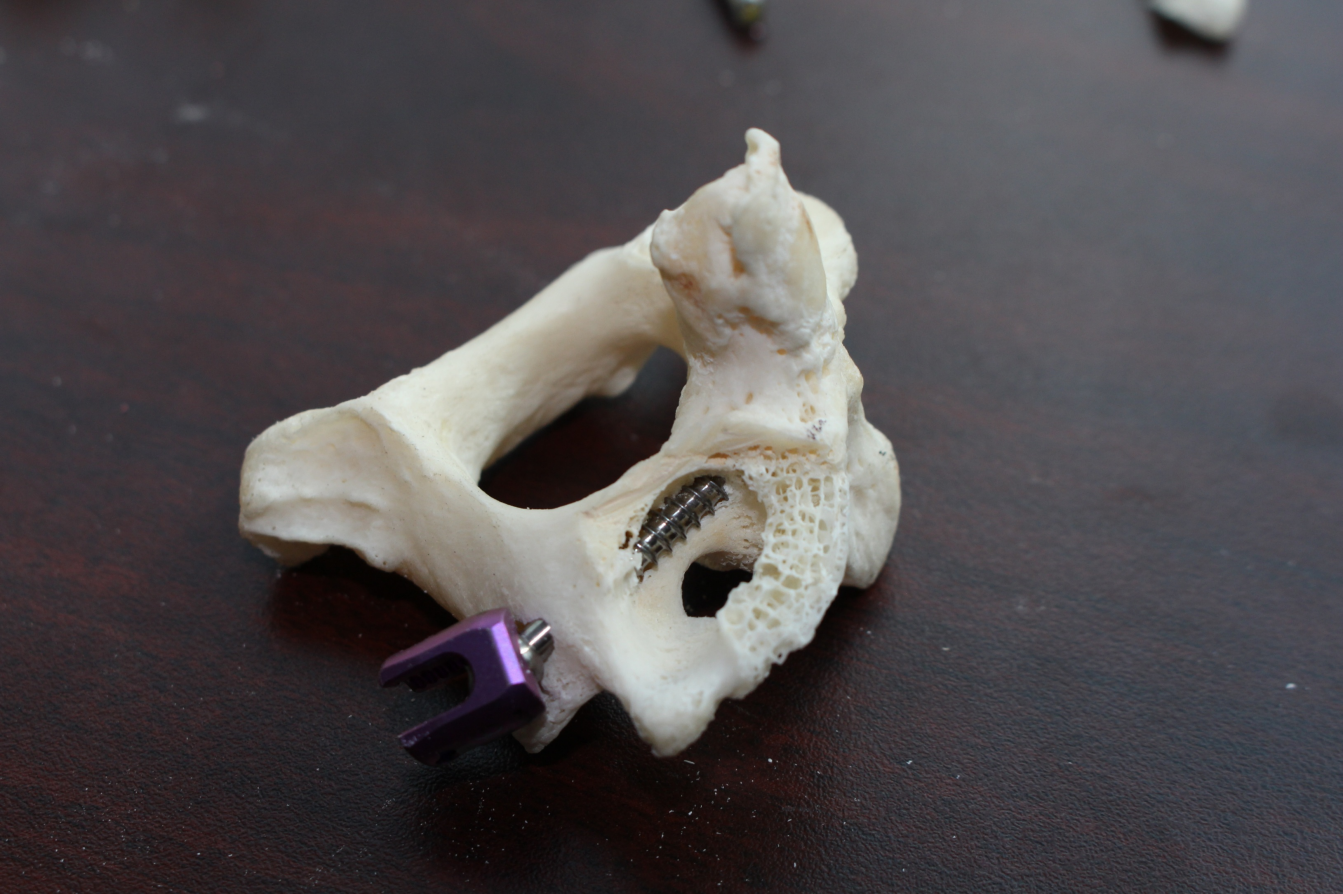
The superior articulating surface of C2 has been cut off, exposing the trajectory of the C2 pedicle screw and the proximity of the pedicle screw to the transverse foramen.

Utilizing dry vertebrae, the superior articulating surface of C2 is cut off at its lateral point of attachment with a high speed drilling disc (Dremel rotary tool, Robert Bosch Tool Corporation, IL, USA), exposing the C2 pedicle and the entirety of the C2 transverse foramen as shown in Figure 1.

**Figure 2:**
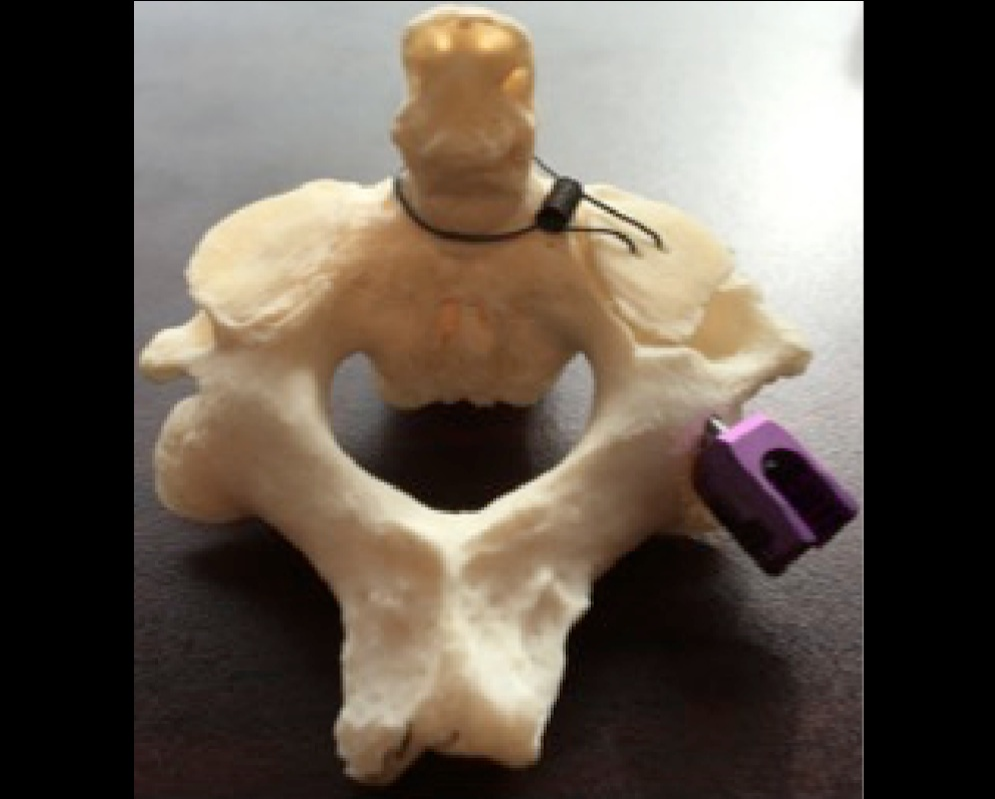
The superior articulating surface of C2 is hinged off the dens and flapped back into place, allowing for free-hand placement of the C2 pedicle screw.

This provides the trainee with a better appreciation for the feel of the pedicle screw as it traverses cortical and cancellous bone, while having clear visual feedback of the position of the pedicle screw relative to surrounding structures in real time. The cut off superior articulating surface of C2 can be hinged off the odontoid process with a small metal spring as shown in Figure 2.

**Figure 3:**
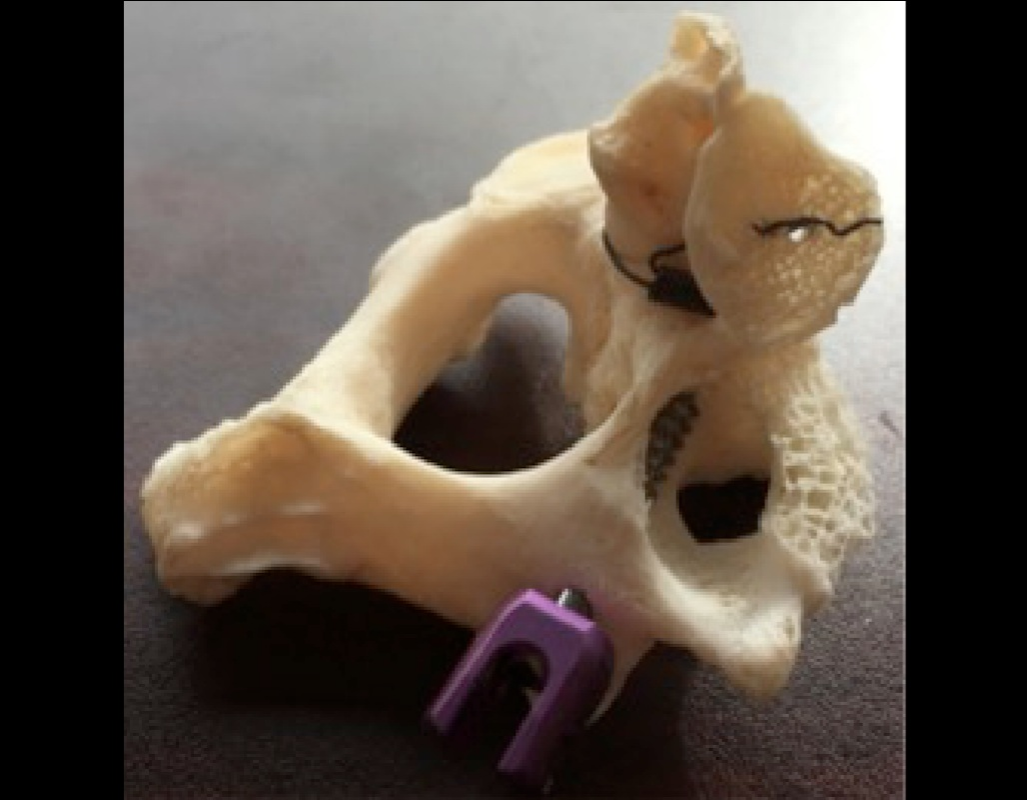
The superior articulating surface of C2 is lifted up to evaluate the trajectory of the pedicle screw and its proximity to the transverse foramen.

This allows for practice placement of the C2 pedicle screw by the trainee based on the landmarks and trajectory described above, as shown in Figure 3.

**Figure 4:**
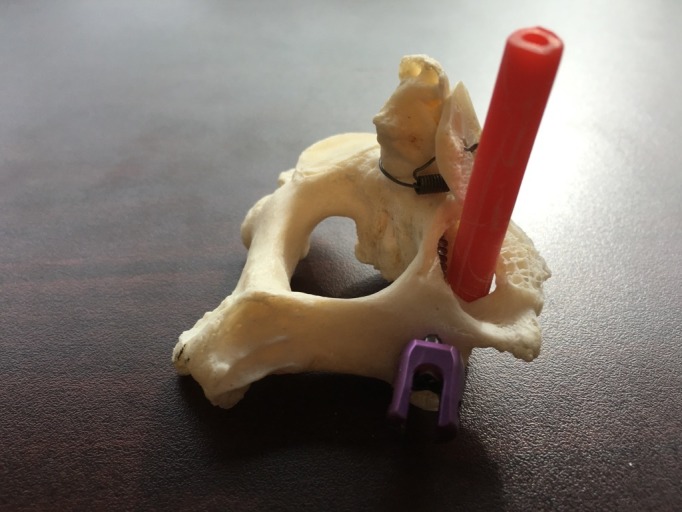
A red tube representing the vertebral artery traversing the C2 transverse foramen shows the proximity of the C2 pedicle to the vertebral artery.

The placement of the screw can then once again be evaluated by lifting the hinged superior articular process and assessing the proximity of the pedicle screw to surrounding structures such as the vertebral artery, which is delineated with a red plastic tube in Figure 4.

This can be repeated as often as necessary until the trainee is comfortable with the technique.

## Conclusions

This cheap and simple teaching aid provides a much needed avenue for preparing trainees for real-life C2 pedicle screw placement while avoiding the associated risks of intraoperative training or the usually prohibitive cost of providing cadavers for training residents and fellows. It can also easily be applied to other technically challenging spinal instrumentation procedures such as C1 lateral mass and thoracic pedicle screw placement.
